# Efficacy of Phage Therapy in Controlling Rabbit Colibacillosis and Changes in Cecal Microbiota

**DOI:** 10.3389/fmicb.2017.00957

**Published:** 2017-05-29

**Authors:** Jian Zhao, Yan Liu, Chenwen Xiao, Shaojie He, Huochun Yao, Guolian Bao

**Affiliations:** ^1^College of Veterinary Medicine, Nanjing Agricultural UniversityNanjing, China; ^2^Institute of Animal Husbandry and Veterinary Science, Zhejiang Academy of Agricultural SciencesHangzhou, China

**Keywords:** bacteriophage, phage therapy, rabbit, colibacillosis, microbiota

## Abstract

Phage therapy is a valid weapon that we could use to fight against pathogens. Bacteriophages kill bacteria and self-proliferate in the digestive tract. Furthermore, it was assumed that phage therapy could preserve the existing gut microbiota. In this study, 45 rabbits were equally divided into three groups after they were orally inoculated with pathogenic *Escherichia coli* to induce gut infection. Each group was treated with bacteriophage ZRP1 (Group P), ciprofloxacin lactate (Group A), or phosphate-buffered solution (PBS) (Group N). Another 15 healthy rabbits composed the control group (Group C). The body weight gain decreased significantly, but the white blood cell (WBC) count, especially the percentage of large WBCs, and the serum endotoxin levels increased significantly after infection. The result of microscopic examination of the ileum showed that *E. coli* ZR1 adhered to villi and caused hemorrhage inside the villi. Groups P and A rabbits recovered after treatments, and both bacteriophage and antibiotic treatment significantly decreased the *eaeA* gene concentration in cecal contents. The microbiota in cecal contents changed in infected rabbits that were treated with PBS. The relative abundance of *Clostridiales* and *YS2* decreased but the relative abundance of *Enterobacteriales* increased significantly. According to the principal components analysis, the microbiota of Groups P and C rabbits were similar to one another in type and relative abundance but different from those of Groups N and A rabbits. The results demonstrated that oral administration of bacteriophage can cure gut infection with minimal impact on the cecal microbiota.

## Introduction

Colibacillosis, caused by pathogenic *Escherichia coli* and characterized by diarrhea or systematic infection, is a common bacteriosis and generates huge economic losses in the animal husbandry industry. In the last two decades, researchers have investigated alternative treatment methods for colibacillosis because of the antibiotics residues and the increasing concern over drug-resistant bacterial strains. Among the promising new agents are bacteriophages, which can specifically lyse host bacterial cells and reproduce themselves approximately one 100-fold, thereby preventing and controlling the disease. Several studies demonstrated that a single orally administrated bacteriophage or bacteriophage cocktail reduced the levels of pathogenic bacteria in the gastrointestinal tract of sheep or mice experimentally infected with *E. coli* (Tanji et al., 2005; Raya et al., 2011) and decreased the rate of diarrhea and the mortality of poultry in farms (Xie et al., 2005).

Rabbits have suffered from colibacillosis caused by enteropathogenic *E. coli* (EPEC) since the beginning of the 1980s (Milon et al., 1999). We recently isolated an atypical EPEC strain (ZR1) and one of its specific bacteriophages (ZRP1) from rabbits. ZRP1 is a member of *myoviridae* family and its genome is a 68201-bp dsDNA molecule with a GC content of 46.16%. The result of blast showed that the DNA shared 96% of identity with the *Escherichia* phage ECML-117 (Query coverage = 93%; *E*-value = 0.0), which is a lytic bacteriophage with a broad target range (Abuladze et al., 2008) and was proved free of bacterial toxin genes, antibiotic resistance encoding genes, and bacterial 16S rRNA genes (Carter et al., 2012). An *in vivo* trial with ZRP1 as an intravenous therapeutic phage showed that it can significantly prolong the survival time of rabbits simultaneously received lethal dose of ZR1 (Zhao et al., 2017). Furthermore, we conducted a trial in rabbits to compare the efficacy of orally administered bacteriophage with antimicrobials and to examine the effect of this phage therapy on the intestinal microbiota profiles.

## Materials and Methods

### Animals Management and Experimental Design

Rabbits in the experiment were raised in cages under controlled temperature (20 ± 2°C) and natural light conditions. Animals had access to water *ad libitum* and were fed 80 g of feed per day per head. The diet mainly comprised hay powder and bran, which provided 15.8 MJ/kg of general energy, 14% crude protein, and 18% crude fiber. The rabbits also received vitamins and trace minerals consistent with the nutrient requirements for growing rabbits. Antibiotics were removed from their diets but anticoccidial drugs were kept.

The study was divided into two stages. In the first stage, 60 New Zealand white rabbits (non-specific pathogen free [SPF]) with an average body weight of approximately 1 kg were allocated to two groups according to their body weights and white blood cell (WBC) counts. The control group (Group C) comprised 15 rabbits free of experimental infection with *E. coli*. Group B was composed of 45 rabbits that were orally inoculated with 1 ml (1 × 10^10^ CFU/ml) of *E. coli* ZR1. In the second stage of the experiment, Group B rabbits were weighed, and their blood samples were collected for WBC count at 3 days post-infection. Based on the body weights and WBC counts, the 45 rabbits were equally divided into three groups: negative control group (Group N), phage-therapy group (Group P), and antibiotic-therapy group (Group A). After grouping, rabbits were immediately orally treated with 1 ml PBS, 1 ml bacteriophage ZRP1 (1 × 10^11^ PFU/ml), and 1 ml ciprofloxacin lactate (20 mg/ml), respectively. Group C rabbits received 1 ml PBS orally each time in order to create the same physical stress in those rabbits. Three days after treatments, all rabbits were weighed, bled for blood samples, and killed for cecal contents collection.

### Histology of Ileum after *E. coli* Experimental Infection

Another two rabbits were sacrificed 3 days after oral infection with *E. coli* in order to investigate whether ZR1 could adhere to the villi of the small intestine. Formalin-fixed ileum specimens were embedded and cut into sections. Sections were stained with hematoxylin and eosin and viewed under an Olympus BX51 light microscope (Olympus [China] Co., Ltd, China).

### Analyses of Blood Samples

Blood samples were taken from the ear vein by pyrogen-free syringes at 1 day pre-infection, 3 days post-infection, and 3 days post-treatment (6 days post-infection). Each time, 40 μl of fresh blood were mixed with heparin sodium; and the WBC counts, as well as the rates of large white blood cells (W-LCR), were examined by an automatic hematology analyzer (pocH-100iV Diff, SYSMEX Shanghai Ltd, China).

Approximately 2 ml of coagulated blood from each rabbit were centrifuged (8,000 *g*; 10 min) for serum in pyrogen-free polyethylene tubes. The lipopolysaccharide (LPS) concentrations were determined by a quantitative chromogenic end-point tachypleus amebocyte lysate (TAL) kit (Chinese Horseshoe Crab Reagent Manufactory Co., Ltd, China) according to the manufacturer’s instruction. Briefly, 100 μl samples were incubated with 100 μl of TAL solutions in non-pyrogen tubes at 37°C for 15 min; then, 100 μl pre-warmed synthetic substrates were added and followed by another 6 min of incubation. Finally, diazo reagents were added; and the absorbance at 545 nm was measured after standing for 5 min. The LPS concentrations were calculated by the standard curve method (Wei et al., 2015).

### Quantitative Real-Time PCR (qRT-PCR) of *eaeA*

DNA from 0.2 g of cecal contents from each sample was extracted using a QIAamp^®^ fast DNA stool mini kit (QIAGEN^®^ Co., Ltd, Germany) according to the manufacturer’s instructions.

The target sequence quantified was a fragment (384 bp) of *eaeA* gene from *E. coli* ZR1. The primers used for the amplification were F-5′ GAC CCG GCA CAA GCA TAA GC 3′ and R-5′ CCA CCT GCA GCA ACA AGA GG 3′ (Luz Maria Chacon et al., 2012). The qRT-PCR was performed on an ABI 7300 system (Applied Biosystems, Thermofisher Scientific Inc., United States). Each experiment was carried out in duplicate with a final volume in each well of 25 μl, which consisted of 12.5 μl SYBR^®^ Premix Ex Taq (Takara Biotechnology [Dalian] Co., Ltd, China), 5 μl DNA samples, 0.5 μl Rox Reference Dye (Takara Biotechnology [Dalian] Co., Ltd, China), 0.5 μl of each primer, and 6 μl ddH_2_O. The program was set following the software wizard (software version 1.4), and 40 cycles were run alternately at 95°C for 5 s and 60°C for 30 s. The specificity of the reaction was checked using a dissociation curve. Pure *eaeA* fragments of known concentration were diluted 10-fold serially to create the standard curve, and the *eaeA* concentration of each sample was calculated by interpolating the *C*_t_ value to the standard curve.

### Analysis of Cecal Microbiota

In each group, three to four DNA samples (see Quantitative Real-Time PCR [qRT-PCR] of *eaeA*) were pooled so that four mixed samples were used for amplifications and the HiSeq sequencing. Universal primers 341F (5′ CCT ACG GGN GGC WGC AG 3′) and 805R (5′ GAC TAC HVG GGT ATC TAA TCC 3′) were used to amplify fragments that covered the V3 and V4 regions of the 16S rRNA genes (Vasileiadis et al., 2012). A HiSeq 2500 system (Illumina Inc., United States) was utilized for sequencing using the PE250 run type. Clean reads were assembled, and aligned tags were clustered at a 97% similarity level into operational taxonomic units (OTUs) referencing the Greengenes 16S rRNA gene database. Microbiota profiles were examined by QIIME for Venn diagram (Bauerl et al., 2014) and R (version 3.3.3) for principal components analysis (PCA) (Combes et al., 2014).

### Statistical Analyses

The statistical software SPSS 17.0 was used to analyze all data except those of microbiota profiles. Least significant difference in one-way analysis of variance was used for multiple comparisons among groups. Square root transformation and log transformation were adopted to render the percentage and qRT-PCR data subject to normal distribution. Additionally, the Kruskal–Wallis *H* test was used to separate differences in bacterial relative abundance among groups.

### Ethics

The study was performed according to the National Institutes of Health Guidelines for the Care and Use of Laboratory Animals (NIH Publication No. 85–23, revised 1996) and was approved by the Animal Care and Use Committee of Institute of Animal Husbandry and Veterinary Science, Zhejiang Academy of Agricultural Sciences.

## Results

### Adhesion of *E. coli* ZR1 to the Ileal Epithelia

**FIGURE 1:**
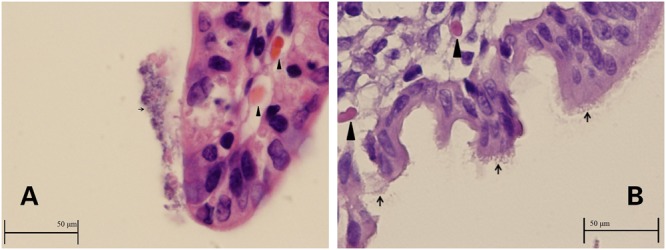
**(A,B)** Images of ileum from rabbits infected with ZR1. Enterocytes and debris covered with *Escherichia coli* (thin arrows) and hemorrhage inside the villus (thick arrowheads).

Intestinal sections from the two rabbits used to investigate ZR1 adherence demonstrated colonization of the ZR1 on villi (**Figures 1A,B**). The integrity of the epithelia was indistinct, and debris attached to *E. coli* was observed, which indicated inflammation and hemorrhage.

### Status of Rabbits in Stage 1

Slight reduction of body weight was observed in the infected group. However, these rabbits exhibited only half of the body weight gain than what was observed in the healthy animals (*P* < 0.05). A significant increase of blood leukocytes, especially neutrophils, occurred after infection in Group B compared to the control group (*P* < 0.05). The endotoxin concentration in the infected rabbits doubled post-infection (*P* < 0.05). Data mentioned above are presented in **Table 1**.

**Table 1:** Body weight, body weight gain, WBC count, W-LCR, and serum LPS concentration of rabbits in different groups before and after *Escherichia coli* infection (Mean ± SD).

	Group C	Group B
Initial body weight (kg)	0.972 @ 0.239	0.975 @ 0.192
Body weight after infection (kg)	1.111 @ 0.236	1.054 @ 0.185
Body weight gain 1 (kg)	0.139 @ 0.016ˆa	0.079 @ 0.084ˆb
Initial WBC count (10^3^/µL)	4.3 @ 1.4	4.3 @ 1.2
WBC count after infection (10^3^/µL)	4.4 @ 1.6ˆa	6.5 @ 2.0ˆb
Initial W-LCR (%)	12.2 @ 2.8	11.8 @ 3.4
W-LCR after infection (%)	9.3 @ 2.6ˆa	16.4 @ 7.8ˆb
Initial Serum LPS (EU/ml)	0.21 @ 0.05	0.22 @ 0.06
Serum LPS after infection (EU/ml)	0.26 @ 0.09ˆa	0.50 @ 0.09ˆb

*Means with no common superscript letter differ significantly (*P* < 0.05).*

### Status of Rabbits in Stage 2

Forty-five infected rabbits were equally divided into three homogeneous groups with very similar weight, WBC or W-LCR values, and serum LPS concentrations. After the treatments, rabbits that received antibiotics had the highest weight gain among all groups (*P* < 0.05) though their average weight was less than that of the control group because of weight-gain lag in the first stage. Rabbits orally inoculated with bacteriophage ZRP1 gained 10 g more than the gain of those without treatment, but the difference was not statistically significant. The WBC number kept increasing except in Group A, but the larger WBCs and the serum endotoxin units decreased at different rates in all groups. The data showed that the leukocyte types and the LPS values of rabbits treated with either the bacteriophage or the antimicrobial returned to normal levels. Additionally, no significant difference in the WBC number was found between groups A and C. Related data are shown in **Table 2**.

**Table 2:** Body weight, body weight gain, WBC count, W-LCR and serum LPS concentration of rabbits with different treatments (Mean ± SD).

	Group C	Group P	Group N	Group A
Body weight after infection (kg)	1.111 @ 0.236	1.054 @ 0.194	1.051 @ 0.193	1.055 @ 0.192
Body weight after treatment (kg)	1.232 @ 0.250	1.178 @ 0.187	1.164 @ 0.188	1.219 @ 0.200
Body weight gain 2 (kg)	0.121 @ 0.051ˆb	0.124 @ 0.034ˆb	0.114 @ 0.017ˆb	0.164 @ 0.034ˆa
WBC count after infection (10^3^/µL)	4.4 @ 1.6ˆa	6.7 @ 1.3ˆb	6.5 @ 1.9ˆb	6.5 @ 2.6ˆb
WBC count after treatment (10^3^/µL)	5.4 @ 1.3ˆa	7.7 @ 1.1ˆb	9.0 @ 2.9ˆb	6.1 @ 1.3ˆa,b
W-LCR after infection (%)	9.3 @ 2.6ˆa	16.9 @ 9.3ˆb	16.7 @ 8.0ˆb	15.7 @ 7.4ˆb
W-LCR after treatment (%)	9.0 @ 2.3ˆa	11.2 @ 2.6ˆa,b	13.0 @ 4.4ˆb	9.4 @ 2.5ˆa
Serum LPS after infection (EU/ml)	0.26 @ 0.09ˆa	0.50 @ 0.07ˆb	0.47 @ 0.08ˆb	0.51 @ 0.11ˆb
Serum LPS after treatment (EU/ml)	0.25 @ 0.06ˆa	0.35 @ 0.09ˆa,b	0.43 @ 0.09ˆb	0.30 @ 0.09ˆa

*Means with no common superscript letter differ significantly (*P* < 0.05).*

### Estimation of ZR1 Amount in the Cecal Residues

Quantitative RT-PCR was performed to estimate the presence of ZR1 by the specific *eaeA* gene and to confirm differences in amount among groups. The *R*^2^ of the standard curve established in this study reached 0.94. The statistical test showed that the *eaeA* gene level of Group N rabbits was significantly higher than in the others (*P* < 0.05). Accordingly, the numbers of tags representing *Enterobacteriaceae* demonstrated the same trend (**Table 3**). A total of 27 OTU types at family *Enterobacteriaceae* level were clustered; and the dominant one was OTU 782953, which is annotated as *Escherichia*/*Shigella* at the genus level. Group N occupied 26 types in the 27 OTUs, while Groups C, P, and A had 3, 6, and 3 types, respectively.

**Table 3:** *eaeA* gene levels and sums of *Enterobacteriaceae* tags in rabbit cecal contents (Mean ± SD or Sum).

	Group C	Group P	Group N	Group A
*eaeA* gene concentration (10^-6^μg/μL)	0.16 ± 0.22^a^	1.07 ± 0.78^a^	16.03 ± 26.58^b^	0.14 ± 0.07^a^
Number of *Enterobacteriaceae* tags	17^a^	74^a^	7265^b^	3^a^

*Means with no common superscript letter differ significantly (*P* < 0.05).*

### Cecal Bacterial Populations of Different Groups

A total of 1,274 OTUs were obtained from all samples (**Supplementary Table S1**). They belonged to 42 orders in 34 classes in 15 phyla. Approximately 50% of the OTUs cannot be identified at the family level, but almost 99.99% of OTUs were clear at the order level. Thus, the data were analyzed and shown at the order level to minimize loss of information.

**FIGURE 2:**
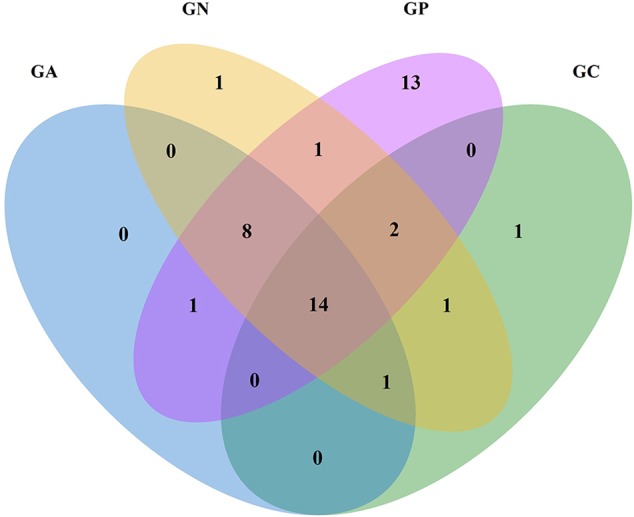
Venn diagram of four groups at the order level according to the classification of the bacterial 16S rRNA V3 and V4 regions. GC, control group; GP, phage-therapy group; GN, negative control group; GA, antibiotic-therapy group.

In the Venn diagram (**Figure 2**), all groups shared 14 orders in common, which accounted for more than 99% of the relative abundance. Another eight common orders were shared by all infected groups. Group P possessed 39 orders including 13 orders in particular, while Groups C, N, and A possessed 19, 28, and 24 orders, respectively.

The relative abundance of *Enterobacteriales* in Group N increased significantly; however, those of the *Clostridiales* and *YS2* significantly decreased. No considerable differences were found among the other three groups. The orders with relative abundance higher than 0.1% in each group are listed in **Table 4**.

**Table 4:** Influence of different treatments on the bacterial core order relative abundance (%) in all groups (Mean ± SD).

	Group C	Group P	Group N	Group A
*Clostridiales*	76.35 @ 5.81ˆa	78.96 @ 2.56ˆa	64.98 @ 8.51ˆb	72.69 @ 5.91ˆa,b
*Bacteroidales*	13.02 @ 3.49	10.23 @ 2.05	18.41 @ 7.04	18.48 @ 4.93
*YS2*	5.13 @ 1.87ˆa	3.68 @ 1.39ˆa	0.75 @ 0.37ˆb	3.15 @ 2.47ˆa
*RF39*	2.03 @ 2.09	3.32 @ 1.84	1.67 @ 0.38	1.97 @ 1.99
*Verrucomicrobiales*	1.43 @ 1.43	2.14 @ 2.11	3.74 @ 2.71	1.55 @ 1.64
*Campylobacterales*	1.44 @ 1.55	0.66 @ 0.57	0.54 @ 0.58	1.13 @ 0.73
*RF32*	0.23 @ 0.17	0.30 @ 0.47	0.01 @ 0.00	0.15 @ 0.07
*Burkholderiales*	0.14 @ 0.10	0.17 @ 0.08	0.27 @ 0.10	0.21 @ 0.12
*Bacillales*	0.001 @ 0.002	0.15 @ 0.22	0.004 @ 0.004	0.04 @ 0.06
*Enterobacteriales*	0.02 @ 0.02ˆb	0.11 @ 0.12ˆb	9.31 @ 12.26ˆa	0.01 @ 0.00ˆb
*Sphingobacteriales*	–	0.001 @ 0.002	0.13 @ 0.21	0.02 @ 0.04
*Flavobacteriales*	–	0.02 @ 0.03	0.03 @ 0.03	0.18 @ 0.31
*Desulfovibrionales*	0.03 @ 0.04	–	0.003 @ 0.003	0.35 @ 0.60

*Means with no common superscript letter differ significantly (*P* < 0.05).*

**FIGURE 3:**
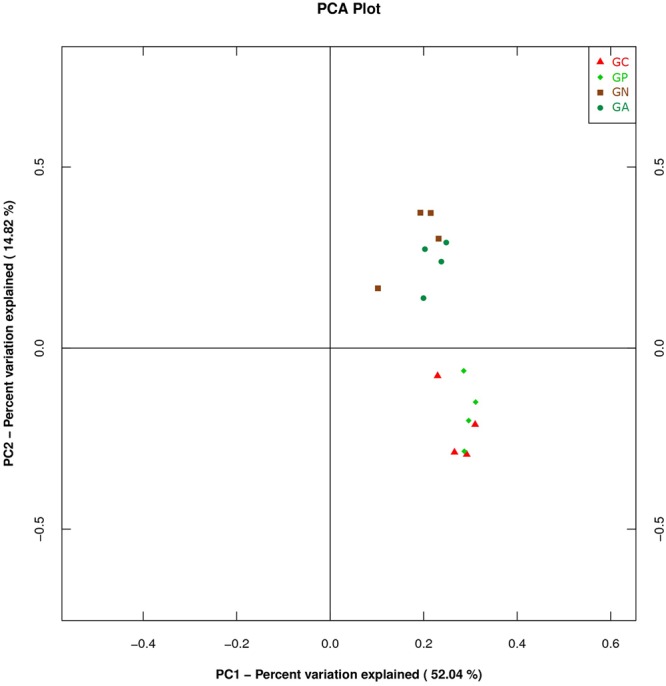
Principal component analysis (PCA) plot based on the first and second PC of bacterial community taxonomic profiles from 16 mixed cecal samples in four groups. GC, control group; GP, phage-therapy group; GN, negative control group N; GA, antibiotic-therapy Group.

**FIGURE 4:**
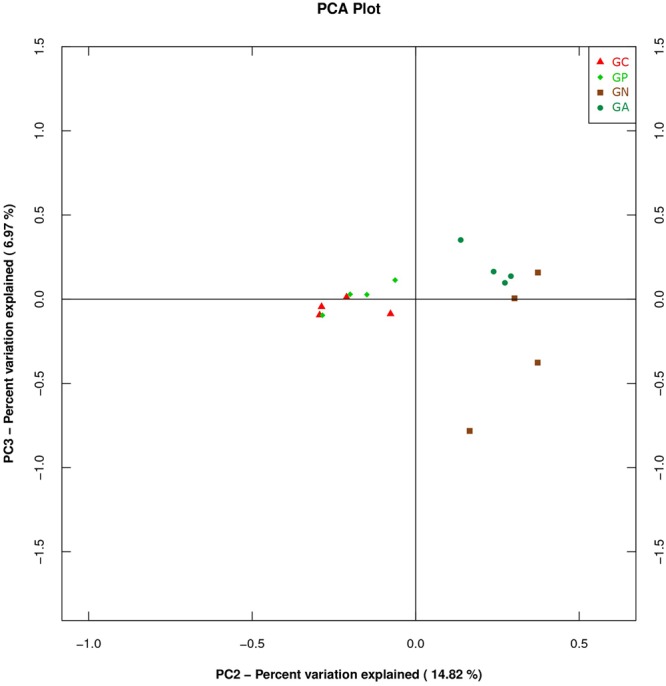
Principal component analysis (PCA) plot based on the second and third PC of bacterial community taxonomic profiles from 16 mixed cecal samples in four groups. GC, control group; GP, phage-therapy group; GN, negative control group N; GA, antibiotic-therapy Group.

A PCA was carried out on 16 taxonomic profiles and tended to separate all samples into two clusters as demonstrated on the PC1–PC2 plot, in which PC1 and PC2 explained 52.04 and 14.82% of the total variation. One cluster comprised Groups C and P, and the other cluster consisted of Groups N and A (**Figure 3**). The main differences in PC2 values indicate that the cecal microorganisms of the two clusters were compositionally distinct from each other in the second principal component. The PC2 was positively correlated to *Burkholderiales* but negatively correlated to *RF32* and *YS2*. That is to say, the high level of *Burkholderiales* and the low level of *RF39* and *YS2* of Group N or Group A raised their scores in the PC2 axis. Furthermore, the slight difference in PC1 value between the two clusters was mainly caused by the variations of *Clostridiales* (positive) and *Bacteroidales* (negative). The PC3 which explained 6.97% of the total variation showed a negative correlation with *Enterobacteriales*. So the significantly high percentage of *Enterobacteriales* in Group N lowered its value in PC 3 axis (**Figure 4**).

## Discussion

Atypical EPEC causes intestinal lesions characterized by attaching and effacing (A/E), and the intimin encoded by the attaching and effacing gene (*eae*) is thought to contribute to the attachment and destruction of microvilli (Hernandes et al., 2009). In our study, histopathological features in the ileum were similar to those previously described (Al-Mamun et al., 2013). Normal intestinal barriers are breached by pathogenic *E. coli* colonization; then bacteria and their products, such as LPS, infiltrate into blood and recruit polymorphonuclear (PMN) leukocytes. Moreover, as reported previously (Gallois et al., 2007; Al-Mamun et al., 2013), growth performances of rabbits are retarded by the infection. And, in our case, the retardation was caused by the decrease in the feed conversion ratio as the feed supply was constant. LPS induces an inflammatory response from animal immune systems by binding to toll-like receptor 4. Intraperitoneal injection of *E. coli* LPS has been used to stimulate an inflammatory state and trigger the increase in WBCs 24 or 48 h after the injection (Brecchia et al., 2010, 2014). In our study, the elevation of blood endotoxin levels and the increase in WBC number were also observed in infected rabbits. In additional, the rise in W-LCR indicated that neutrophils responded quickly to the infection during the acute phase of inflammation.

Phage therapy predates the discovery of penicillin and has been investigated as an alternative to antibiotics recently. Bacteriophages are harmless to animals, and their efficacy is superior to that of antibiotics in certain respects (Smith and Huggins, 1982; Bull et al., 2002; Zhang et al., 2015). In our experiment, application of ZRP1 successfully reduced the *E. coli* ZR1 load in the intestine. Either the *eaeA* gene abundance or the *Enterobacteriaceae* tag numbers were substantially lower in treated as compared to in untreated rabbits. qRT-PCR is more sensitive and specific than conventional culture method for diagnosis and quantification (Bischoff et al., 2005). Therefore, the *eae* gene was selected to identify certain EPEC strain in feces using this method (Al-Mamun et al., 2013). Theoretically, the *eaeA* gene should not have been detected in the control group. One possible reason we detected in our control group is because the rabbits we used in the experiment were non-SPF animals and might have carried very few bacteria with the *eae* gene prior to the start of the experiments. With the elimination of ZR1 in the gastrointestinal tract, serum LPS concentrations and the W-LCR values decreased accordingly, hence the body weight gain recovered after the phage treatment. Ciprofloxacin lactate was more powerful than we expected in treating ZR1 *in vivo*. This treatment nearly wiped out the pathogenic *E. coli*; and a consequent compensatory growth was observed in the rabbits. We assumed that the inferior phage therapy efficacy could be the results of using an insufficient phage dose. The influence of bacteriophage-to-bacterium ratio was reviewed (Hraiech et al., 2015); and a previous study concluded that a larger phage dose was superior in passive treatments (Tsonos et al., 2014). The sensitivity of ZR1 to ciprofloxacin lactate may play a key role as well. The studies we cited above (Smith and Huggins, 1982; Bull et al., 2002; Zhang et al., 2015) did not mention the relationships between the pathogens and the antibiotics, but we chose ciprofloxacin lactate by antimicrobial susceptibility testing. Nevertheless, bacteriophages demonstrate a virtue not apparent in antibiotics: phages self-replicate rather than being metabolized *in vivo*. Thus, dosing bacteriophages repeatedly is unnecessary (Mandal et al., 2014). In our research, numerous active bacteriophages (>10^5^ PFU/g feces, data not shown) were found 3 days after the treatment. Taken the coprophagy of rabbits into account, these bacteriophages had a great chance to re-enter the digestive tract. Moreover, these bacteriophages might play a role in limiting host *E. coli* numbers in the environmental reservoir. Thus, the potential benefits of phage therapy may be seen long term.

After birth, rabbits are colonized by a complex and dynamic consortium of microorganisms in the digestive tract; and the bacterial community alteration could be caused by inflammation. In our study, the Venn diagram shows that Groups P, N, and A had eight orders in common. This may be attributed to the altered intestinal environment caused by the experimental infection of *E. coli* ZR1, creating niches for those bacteria to grow. However, the emergence of 13 particular orders in cecal contents of phage-therapy rabbits is still a mystery.

Using a highly effective sequencing technique, the representation of some cecal bacteria was established. Except *Cyanobacteria*, four phyla (*Firmicutes, Bacteroidetes, Tenericutes*, and *Verrucomicrobia*) in the top five and their relative abundances in the cecum of healthy rabbits were very similar to those previously reported (Bauerl et al., 2014). Likely, in rabbits infected but untreated, the increase of *Enterobacteriales, Bacteroidales*, and *Verrucomicrobiales* accompanied by a decrease of *Clostridiales* were observed, too (Bauerl et al., 2014). The results indicated a negative correlation between *Clostridiales* and the pathogens when the intestines of healthy rabbit were infected. Nevertheless, Gut bacteria plays an important role in food fermentation and therefore in health status. The rabbit’s cecal microbial composition was dominated by *Ruminococcaceae* (in the order *Clostridiales*) which succeeded *Bacteroidaceae* (in the order *Bacteroidales*) after weaning to adapt solid feed and such alteration reduced the mortality of weaned kits (Combes et al., 2014).

Studies using PCA for analyzing gut microbiota of rabbits receiving different treatments are very few. PCA is a method to covert a lot of observations into a set of linearly uncorrelated variables. Basically, the closer the dots on the PCA plot are, the more homologous they are. In our study, samples from Groups C and P had PCA plot points that appeared in a cluster. We assumed that the narrow antibacterial spectrum of bacteriophage ZRP1 preserved the existing microbiome. On the contrary, ciprofloxacin lactate, a broad-spectrum third-generation fluoroquinolone, might be harmful to *YS2* (in the phylum *Cyanobacteria*) because ciprofloxacin is toxic to *Cyanobacteria* (Ebert et al., 2011). Antibiotics are widely used in animal disease treatment and prevention or feed efficiency improvement, but they destroy pathogens and commensal bacteria simultaneously. A short-term antibiotic treatment can shift the population structure of the microbiota and alter bacterial physiology, such as reduction of the amount and diversity of microbes, losses in the function of metabolism and the modulation of the immune system (Ferrer et al., 2017). Therefore, antimicrobial agents with minimal side-effects on commensal bacteria are badly needed (Toutain et al., 2016).

Normally, each individual phage has a very narrow host range that will benefit microbial community by affecting only a relatively small subset of bacteria (Dalmasso et al., 2014). The advantage of phage therapy is to specifically target pathogens while not to perturb the normal microbiota. If the normal gut microbiota is not disrupted in a designed phage therapy, they will provide protection to the gut from disease by stimulating the recovery of the immune response to pathogens (Belizario and Napolitano, 2015; McCarville et al., 2016).

Up to now, little was revealed about the role of phage therapy in shaping the gut microbiota and its possible influence on health. The potential impact of phages on bacterial population also relies on the opportunity of encounters between lytic phages and their host *in vivo* (Dalmasso et al., 2014). The result of our work suggested that the postulate that phage therapy has little impact on the gut bacteria ecology is well-founded.

## Conclusion

Rabbits orally infected with pathogenic *E. coli* can be cured by a single specific phage therapy. The phage removed nearly all host cells *in vivo* and had few effects on other bacteria. However, comparing to those received antibiotic treatment; phage-therapy rabbits had a little higher residual ZR1 load in cecum contents at the end of the 3-day trial. It deserves further study using a longer period to see whether phage therapy is as effective as antibiotic treatment.

## Author Contributions

Conceived and designed the experiments: JZ, YL, HY, and GB. Performed the experiments: JZ, CX, and SH. Analyzed the data: JZ and YL. Contributed reagents/materials: CX. Contributed to the writing of the manuscript: JZ and YL. Revised the manuscript: GB.

## Conflict of Interest Statement

The authors declare that the research was conducted in the absence of any commercial or financial relationships that could be construed as a potential conflict of interest.

## Supplementary Material

TABLE S1 **Total OTUs and tags of 16 mixed samples in four groups**.
